# Co-designing accessible digital mindfulness for veterans with spinal cord injury: protocol for developing and testing VA CALM-SCI for virtual reality

**DOI:** 10.3389/fdgth.2026.1923501

**Published:** 2026-10-08

**Authors:** Hilary Touchett, Kathryn Kanzler, Zina Trost, Jan Lindsay, Felicia Skelton

**Affiliations:** 1 Center for Innovations in Quality, Effectiveness and Safety (IQuESt), Michael E. DeBakey VA Medical Center, Houston, TX, United States; 2 Department of Medicine, Baylor College of Medicine, Houston, TX, United States; 3 South Central Mental Illness Research, Education, Clinical Center, a Virtual Center, Houston, TX, United States; 4 VHA Office of Rural Health’s Veterans Resource Center in Salt Lake City, Salt Lake City, UT, United States; 5 Department of Psychiatry and Behavioral Sciences, Baylor College of Medicine, Houston, TX, United States; 6 Department of Psychological and Brain Sciences, Texas A & M University, College Station, TX, United States; 7 Department of Physical Medicine and Rehabilitation, Baylor College of Medicine, Houston, TX, United States

**Keywords:** accessibility, co-design methods, equitable digital health, mindfulness, spinal cord injury (SCI), veterans, virtual reality (VR)

## Abstract

**Introduction:**

Virtual reality (VR)-based mindfulness programs are gaining traction as innovative, engaging, and low-cost tools to address chronic pain, depression, and anxiety. However, these programs are often designed for neurologically normative populations while lacking accessibility for people with disabilities, such as spinal cord injury (SCI). This protocol describes a participatory co-design approach adapting the Department of Veterans Affairs Compassionate Awareness Learning Module (VA CALM) content for individuals with SCI to improve access and accessibility and then evaluate the feasibility and acceptability of delivering this program via VR to enhance effectiveness and engagement.

**Methods and analysis:**

This 2-phase formative study uses the Method for Program Adaptation through Community Engagement (MPACE) to guide our co-design process. In Phase 1, 10 Veterans with SCI will complete VA CALM to identify needed adaptations. Quantitative measures will assess mindfulness, pain (interference, catastrophizing, and acceptance), depression, anxiety, and quality of life (QOL). Qualitative data include field notes, facilitator reflections, and focus groups. A 10-member steering committee of clinicians and Veterans will review findings and adjudicate adaptations. In Phase 2, preliminary VA CALM–SCI modules will be delivered via a VR platform. Feasibility and acceptability will be evaluated for usability, user experience, and embodiment alongside qualitative feedback.

**Discussion:**

This study advances equitable digital health by co-designing accessible mindfulness content for SCI and testing a VR delivery model that may reduce travel burden, enhance engagement, and expand clinical capacity. Findings will inform future efforts in developing and testing this adapted program and reinforce the value of prioritizing accessibility and inclusion when developing future healthcare innovations.

## 1 Introduction

### 1.1 Chronic pain, mental health, and mindfulness in SCI

Anxiety, depression and chronic pain are associated with poorer quality of life (QOL) after spinal cord injury (SCI); and there is a high prevalence of these comorbidities among those with SCI. Nearly 1 in 3 individuals with SCI is diagnosed with anxiety after injury (1), and nearly 1 in 5 are depressed (2). Additionally, an estimated 70% of individuals with SCI struggle with chronic pain (i.e., pain lasting more than 3 months) (3). Chronic pain negatively impacts QOL (4), life satisfaction (5), and social functioning/participation for individuals with SCI (6). In fact, a qualitative study among individuals with SCI characterized having chronic pain as “the battle for ultimate agency in life”(7, p. 2207). Having comorbid mental health disorders such as anxiety and depression is associated with higher reported pain scores (5, 8, 9) and lower levels of functional independence (5, 10). Chronic pain negatively impacts psychological well-being and functional independence (11, 12). Current evidence-based approaches indicate that treatments focusing on psychosocial well-being (e.g., mindfulness) are more effective in improving pain-related QOL than pharmaceutical pain intervention alone among individuals with SCI (13, 14).

Nonpharmacological solutions for chronic pain are vital for several reasons: (1) Neuropathic pain can be refractory to pharmaceutical management in SCI (13), and the chronicity of pain can lead to habituation and upregulation of pain receptors. (2) U.S. Veterans with SCI tend to require intensive medical management and be on multiple prescription drugs (15). (3) Polypharmacy creates complexity for clinicians in balancing drug interactions and side-effect profiles and leads to confusing dosing schedules that may lead to patient mismanagement of medications (16). (4) Lastly, many commonly prescribed pain medications, particularly anticonvulsants, anxiolytics and opioids, are central nervous system depressants, which negatively impact physical and cognitive function and contribute to gastrointestinal and genitourinary issues that impact continence and QOL in this population (16).

However, from a biopsychosocial viewpoint, pain is a multidimensional construct that encompasses intensity and debility. Understanding pain interference moderates the impact of chronic pain on QOL and social participation (12, 17, 18). Pain interference refers to the level of perceived intrusion that chronic pain has on overall functioning and well-being. Pain interference explains greater variance in quality-of-life scores than pain type or pain intensity (19). Pain interference is closely linked to pain catastrophizing, which is the propensity to focus on pain sensations and to negatively qualify one's ability to cope with pain. Pain catastrophizing is associated with greater pain intensity, psychological distress, pain-related disability, and pain interference in individuals with SCI, which impacts QOL (19).

However, acceptance of chronic pain mitigates pain interference and is associated with higher QOL, physical functioning, and social participation (20). Pain acceptance is characterized by a willingness to experience pain but still engage with desired and valued activities (21). As a robust predictor of adjustment to chronic pain, it has been shown to successfully buffer increases in pain intensity (21).

Mindfulness is a proven strategy that increases pain acceptance and QOL. Mindfulness is the practice of being aware of the present moment in a sustained, accepting and nonjudgmental way (22). Mindfulness has shown efficacy in reducing symptoms of anxiety and depression, which are associated with higher pain scores and lower levels of functional independence (5, 10). Being less mindful is linked to pain catastrophizing, which increases avoidance of activities that may provoke pain, thereby decreasing overall function and participation (23). Programs centered around mindfulness are effective nonpharmacological interventions that work by promoting pain acceptance, which reduces pain catastrophizing and pain interference, thereby improving overall QOL (20).

Mindfulness-based interventions are effective in the SCI population because they improve depression, anxiety and general mental health status (14, 24). Mindfulness is broadly applicable to individuals with SCI and chronic pain of all etiologies because, rather than targeting the direct cause of the pain, mindfulness fosters better coping skills to reduce the level of impact pain has on daily functioning.

Furthermore, SCI creates a physiologic disconnect between the mind and body. Impaired communication between the brain and the peripheral nervous system impacts motor, sensory and autonomic functions such as breathing, urination and defecation. Mindfulness addresses the inherent disconnect between mind and body that is unique to individuals with neurological alterations like SCI. While many populations benefit from strengthening the psychological mind-body connection, individuals with neurological alterations like SCI have the added barrier of physiologic disconnection. Mindfulness may impact rehabilitation in several ways. As mentioned previously, mindfulness has benefits for psychological well-being after SCI, particularly in the context of comorbid depression, anxiety and chronic pain (14). Additionally, there is increasing evidence that physiologic changes in the brain occur when mindfulness practices are consistently used, particularly in the centers of the brain responsible for emotion regulation and self-awareness (25, 26). This is especially important considering the role of neuroplasticity in recovery. Neuroplasticity involves the process of reorganization or changes in neuronal activation patterns. Historically, plasticity and reorganization in adults were thought to be confined to a short period of time after an injury. However, current research supports that plasticity and neuronal reorganization occur throughout the lifespan, although at a slower place, provided there is “functional exercise” to promote plasticity (27, 28). With this in mind, mindfulness practices that promote intentionality, body scanning and awareness may be an area of future developmental work to determine whether there is a potential impact on neuroplasticity (29, 30).

### 1.2 The VA CALM program

The Department of Veterans Affairs (VA) Compassionate Awareness Learning Module (VA CALM; (24, 31, 32) is a mindfulness program developed and tested specifically for Veterans that is currently delivered nationally as part of the Whole Health initiative within VA. Whole Health is a program within VA that uses a holistic approach to supporting Veteran health and well-being by providing a range of evidence based complementary and alternative interventions to support wellness. Based on traditional mindfulness-based stress reduction techniques, VA CALM can be delivered in person or via video telehealth by a trained instructor (training courses are open to all disciplines) in a group setting in weekly 1 h sessions over 9 weeks. The program delivers practical information in concrete, easily digestible blocks. The group structure may benefit self-efficacy and social participation by providing a space to normalize pain experiences and promote social connectedness, which is particularly important for individuals with limited community mobility and limited interaction opportunities with SCI peers.

SCIs create alterations in sensation, motor control and proprioception that can limit the accessibility and fit of mindfulness program content and activities for Veterans with SCI. Although the VA CALM program has demonstrated major benefits (24, 31, 32), mindfulness activities were designed for “neurologically normative” populations, without sufficient modifications and accommodations for those with neurologic alterations. For example, a module focused on mindful eating might fall flat for a Veteran who receives nutrition through enteral feeding tubes. To ensure Veterans with SCI are not excluded from programs like this, modified activities such as using flavored oral swabs or having the Veteran focus on feeling fullness when receiving tube feeding should be developed to teach mindfulness principles. A series of qualitative studies found that poorly tailored activities can be triggering and off-putting (33), noted significant barriers impacting the adoption of mindfulness after injury and found 10 core areas requiring adaptation for neurological impairment (34). Yet, more work is needed to determine what adaptations for VA CALM are needed for Veterans with SCI.

### 1.3 Virtual reality (VR) as a delivery modality

VR facilitates immersion, presence, and embodiment, which can enhance the mind-body connection. When seeking to improve the mind-body connection, VR benefits individuals with SCI with chronic pain, because it improves embodiment (35). Embodiment represents integration of the mind and body by intentionally training self-awareness, using all senses available. VR may aid embodiment by creating an immersive experience that provides multisensory processing to increase a sense of body ownership. This is achieved through visual, auditory, and haptic (tactile) feedback delivered through the device. View, sounds, and strength/location of tactile feedback are modified through application features and wearable accessories to promote a multisensory experience for individuals with neurological alterations. Due to innovations like these, VR modalities are gaining traction across the medical community as engaging, low-cost tools that provide a highly immersive and customizable treatment modality for SCI populations (36, 37). Although VR has been used for mindfulness training and chronic pain treatment with moderate success, there is limited research leveraging the strengths of these approaches combined in the SCI population; and further research is needed (35, 36).

### 1.4 Rationale for adaptation and co-design

The proposed program is novel in several ways:

1. VA CALM was designed for neurologically normative populations. Adapting this program by developing a list of strategies, tools and accommodations that make this intervention more accessible for Veterans with SCI (i.e., VA CALM-SCI), gives it broader applicability and potentially improves impact.
2. VR facilitates mindfulness through immersion and embodiment. VA CALM is currently delivered in person and via video telehealth. Translating VA CALM to a VR platform may improve program outcomes by using a treatment modality that is more immersive, engaging and supportive of mindfulness practices.
3. The use of VR is expanding. VR has predominantly been used in face-to-face settings (e.g., in-person physical therapy sessions) and in self-guided mode, with a standalone product delivered to the individual directly for self-led participation. This program will deliver the intervention in a clinic-to-home set up, allowing clinician-moderated intervention, while providing a more immersive experience than video telehealth and greater accessibility than in-person clinic visits.
4. Combining VA CALM with a VR clinic-to-home delivery modality employs 2 approaches (virtual and group) that expand clinical capacity. Conducting a treatment *group* rather than *individual* treatment allows a clinician to serve multiple individuals in 1 clinic slot, and a clinic-to-home format reduces the need for clinical space and other support while using a highly engaging and desirable modality (VR).

Innovations in digital health technologies like VR provide engaging, effective ways to improve health and well-being (38, 39). In SCI, digital health tools have a vast potential to improve access to health and well-being programs that could improve long-term outcomes for this medically complex population (38). However, despite efforts to address equitable access to digital health, accessibility and fit remains a common constraint of the applications, content, and systems in which these tools are deployed (40). When interventions and programs are developed and tested in normative populations, unintended accessibility barriers often impact intervention, acceptability and efficacy (41, 42).

Research and implementation of digital health in the SCI population lags behind others, particularly for behavioral health interventions (38), creating a disparity for individuals with SCI and other populations with complex disabilities. Improving health outcomes requires more than expanding availability; interventions must be adapted to the disability context (40, 42, 43). Applications, processes, and program content should be tailored to optimize accessibility, ensuring digital tools are easy to understand, easy to use, and salient to the individual needs. Therefore, our team will employ a disability centered co-design protocol to ensure accessibility and contextual fit to deliver and test a VR based VA CALM program tailored for individuals with SCI (41, 44).

### 1.5 Study objectives

The overall aim of this study is to explore the feasibility and acceptability of an SCI-tailored, evidence-based mindfulness group (VA CALM) when delivered using VR, using a mixed-methods approach. To achieve this, the project will employ a 2-phase approach that first evaluates modification needs and makes adaptations to the VA CALM curriculum and then delivers modified modules to explore the feasibility and acceptability of using VR to deliver this programming. The goal of this work is to expand access to care for Veterans with SCI who are at risk of being left out of innovative clinical advancements like VR based mindfulness programming, due to the complexity of their medical condition. Lessons learned may have valuable insights for other motor and sensory impaired populations.

The study objectives are:

1. Identify and create *program modifications*, accommodations, and tools to adapt VA CALM for SCI (VA CALM-SCI), using co-design methods to support access and inclusion for Veterans with SCI.
2. Explore *feasibility* of using VR to deliver VA CALM-SCI to Veterans with SCI (primary outcome).
3. Assess the *acceptability and usability* of using VR to deliver VA CALM-SCI to Veterans with SCI (secondary outcomes).
4. Develop preclinical validation by creating a streamlined process and identifying preferred clinical targets for efficacy testing (exploratory outcomes).

## 2 Methods and analysis

### 2.1 Study design

This study will use a sequential 2-phase, mixed-methods approach to conduct a formative evaluation of the VA CALM program to tailor content for the needs of individuals with SCI. Phase 1 consists of providing the unadapted VA CALM content to a group of Veterans to gather feedback and insights on needed modifications. Next, a steering committee guided by the Method for Program Adaptation through Community Engagement (M-PACE) process will recommend changes in content and adaptive strategies, and recommend tools to address issues identified during this phase (45), using the Framework for Reporting Adaptations and Modifications (FRAME) to guide systematic documentation of changes to the evidence-based practice (46, 47). Phase 2 will then explore the feasibility and usability of using VR to deliver VA CALM-SCI by delivering 2 adapted modules over VR to gather Veterans' feedback on adapted content and the use of VR (as compared to video telehealth) to facilitate these sessions. Due to the developmental nature of this work, participation in this study will not impact participation in ongoing standard care.

### 2.2 Phase 1: program adaptation

#### 2.2.1 Participants

##### 2.2.1.1 Inclusion criteria

- Age of at least 18 years (no upper limit was set for this pilot study to ensure inclusiveness of all ages),
- Documented diagnosis of SCI based on American Spinal Injury Association Impairment Scale (AIS) score in VA Spinal Cord Injury and Disorder Registry
- Documented history of chronic pain lasting ≥3 months per electronic health record

##### 2.2.1.2 Exclusion criteria

- Lack access to high-speed internet
- Lack minimal computer proficiency (e.g., able to check emails, read news, attend virtual meetings).
- Unable to put on/take off VR equipment either independently or with caregiver support
- Unable to speak clearly and independently for focus groups
- Unable to consent for research or medical care

##### 2.2.1.3 Recruitment

Approximately 10 participants will be recruited from the Houston VA SCI hub through chart reviews and clinician referral for initial eligibility based on inclusion criteria. Mailouts include information about the study, directions to opt-out of contact by phone from the study team, and a consent form. If eligible individuals do not opt out within 2 weeks after mailing, a member of the study team will attempt to contact them by phone up to 3 times before removing them from the recruitment contact list. During telephone contact, interested individuals will be provided with a brief description of the study and screened for eligibility based on exclusion criteria. If found to be eligible, interested individuals are invited to review the consent form with the study team member over the phone and then directed to sign/return the consent form via mail or provide their email address for a team member to send the form via Docusign to improve accessibility.

##### 2.2.1.4 Sample characteristics

Given the heterogeneity of the SCI population, the following data will be abstracted from the electronic health record: neurological level of injury and AIS grade, time since injury, pain phenotypes (e.g., neuropathic, nociceptive, mixed), presence and severity of spasticity, documented cognitive impairments, active psychiatric diagnoses, and active pain treatment(s). Due to the small sample size and the formative nature of this work, the study is not powered to stratify outcomes; however, the data will be used to contextualize feasibility findings and inform eligibility and stratification planning for a future randomized controlled trial (RCT).

#### 2.2.2 Intervention: delivery of unmodified VA CALM

Phase I involves the delivery of VA CALM over VA Video Connect, a video telehealth platform used in the VA, in the original, unmodified state to gather participant feedback on needed changes to support accessibility and inclusion for individuals with SCI. Participants will attend 9 weekly sessions, delivered by a SCI physician trained to deliver VA CALM. Sessions will last approximately 60–90 min. Modules cover a range of mindfulness topics and essential skills. Table 1 provides a schedule and overview of sessions. Participants will receive a VA CALM Handbook, which includes exercises to practice skills.

**Table 1:** VA CALM schedule and content.

#	Module and topic	New skill homework (each week adds new skills)
0	Program introduction	N/A
1	Why mindfulness	Perform a body scan.
		Practice mindful eating exercise.
2	Perception	Mindfully complete an activity of daily living (dressing, bathing, grooming, eating).
		Complete pleasant events calendar.
3	What makes something a Pleasant Experience?	Practice creating a breathing space.
		Complete unpleasant events calendar.
4	Being stuck	Practice awareness of stressors, awareness of feeling stuck/numb.
		Reflect on sensations and thoughts/emotions.
5	Getting unstuck	Practice loving kindness.
		Complete the difficult communication calendar.
6	Communication	Practice breath, body, emotions, thoughts, choiceless awareness.
		Explore mindful listening.
		Reflect on communication styles (assertiveness/empathy in difficult communications).
7	Mindfulness in daily life	Experiment practicing activities without the audio guide.
		Think about what you need to support a continued daily practice.
8	Today is the first day of the rest of your life	Continue with what has been working for you.
		Consider finding a group to maintain your mindfulness practice.

#### 2.2.3 Data collection

The primary outcome of interest for this study is the feasibility of using VR to deliver an SCI adapted version of VA CALM to Veterans with SCI. Acceptability and usability of using VR to deliver this program are secondary outcomes that will provide insight on the viability of future development of this work, while clinical measures will be captured as exploratory outcomes to identify ideal clinical targets for future efficacy testing.

##### 2.2.3.1 Quantitative data

In Phase I, quantitative data will focus on using a measure battery administered via VA Redcap to identify potential impacts of the VA CALM program for individuals with SCI; while exploring clinically meaningful changes in pain, mindfulness, depression, anxiety, and QOL. These measures will inform development and refinement of the program to inform an RCT of the fully VR-delivered VA CALM-SCI to streamline processes and reduce participant burden. Table 2 includes descriptions of the measures used in Phase 1 and collection time points. Feasibility benchmarks for Phase 1 include enrollment of ≥70% of participants who were screened and found eligible, retention of ≥70% of enrolled participants through Module 8, AND completion ≥70% of quantitative assessments among retained participants. These thresholds are consistent with other feasibility studies involving individuals with complex medical conditions and disabilities (48, 49).

**Table 2:** Phase 1 quantitative measures and collection timelines.

Outcome	Measure	Length and est. completion	Collection timepoint
Treatment descriptives			
Concomitant pain treatments	Medications used for pain management (e.g., opioids, gabapentinoids, antidepressants, cannabinoids, muscle relaxants) Physical Therapy Psychological Treatments Complementary pain interventions (e.g., acupuncture, massage, supplements)	1 item, 3 min.	T1 Baseline T2 Midpoint (Module 4) T3 Completion (Module 8) T4 Follow-Up (2-weeks post completion)
Exploratory outcomes			
Pain	PROMIS-Pain Numeric Rating Scale (NRS) (50)	1 item, 1 min.	T1 Baseline T2 Midpoint (Module 4) T3 Completion (Module 8) T4 Follow-Up (2-weeks post completion)
Pain- interference	PROMIS-Pain Interference (PROMIS-PI, ShortForm8a) (50)	8 items, 2 min.	
Pain-catastrophizing	Pain Catastrophizing Scale (PCS) (51)	13 items, 3 min	
Pain-acceptance	Chronic Pain Acceptance Questionnaire-Revised (CAPQ-R) (52)	20 items, 5 min.	
Mindfulness	Five Facet Mindfulness Questionnaire (FFMQ) (53) Mindfulness Attention Awareness Scale (MAAS) (54)	39 items, 10 min. 15 items, 4 min.	T1 Baseline T2 Midpoint (Module 4) T3 Completion (Module 8) T4 Follow-Up (2-weeks post completion)
Depression	Patient Health Questionnaire-9 (PHQ-9) (55)	9 items, 2 min.	
Anxiety	Generalized Anxiety Disorder (GAD-7) (56)	7 items, 2 min.	
Quality of life	World Health Organization Quality of Life-Brief (WHOQOL-BREF) (57)	26 items, 7 min	
Feasibility of VA CALM	Number of participants screened; enrolled, drop-outs, session attendance; assessment completion; program completion rates	–	T4 Completion

##### 2.2.3.2 Qualitative data

Given the primary goal to adapt VA CALM for SCI, qualitative data are essential to provide nuanced information regarding contextual issues and needed program modifications. The study will collect qualitative data through field notes collected during each session by the first author (as an observer), facilitator reflections (15 min debriefs after each session), and focus groups conducted with participants consisting of two, 2-h-long semi-structured sessions conducted via video conference. Focus groups will be audio recorded and transcribed verbatim. Table 3 provides a description of data sources and collection points. Data will iteratively inform steering committee agendas and capture issues for further exploration in future focus groups and for triangulation of data during analysis.

**Table 3:** Phase 1 qualitative data and collection timelines.

Source	Description	Collection timepoint
Field notes	Documentation of content, context, and concepts pertaining to participant needs and program implementation (e.g., accessibility issues, etc.)	Weekly during sessions
Facilitator reflections	Documentation of facilitator impressions and observations related to program content and delivery	Weekly after each session
Focus groups	Semi-structured, open-ended questions, assessing content, delivery, and acceptability of the program	Midpoint (Module 4), Completion (Module 8)

##### 2.2.3.3 Characterizing concomitant care

Concurrent treatments (e.g., medications, therapy, and other complementary pain interventions) will be documented via chart review at baseline and assessed via self-report at each quantitative collection timepoint (see Table 2). Due to the developmental nature of this work, participants will not be asked to change or restrict pain treatment as part of their standard care while participating in this study. Changes in treatment during the study period will be used to provide context during data analysis and described descriptively.

#### 2.2.4 Steering committee and co-design process

The study team will assemble a steering committee comprising panel members with expertise in 1 or more of the following: SCI, mindfulness or whole health, and VR. Per the M-PACE process, the ideal steering committee comprises 10–12 individuals; as too few can result in limited perspectives, and too many can impede progress. The steering committee will include members of the research team, a VA CALM facilitator, members of the SCI clinical team (physicians, nurses, physical therapists, occupational therapists, psychologists, VA Whole Health instructors) with expertise in mindfulness and virtual reality programming, and 2–3 Veterans with SCI.

Members of the steering committee will be provided with VA CALM patient and facilitator materials for review and VR headsets for testing. They will meet monthly via video conference to (1) review 1 module of the curriculum; (2) discuss anonymized data collected from participants, focus groups, and field notes; (3) identify areas requiring accommodation; and (4) generate recommendations for strategies, tools, and accommodations to support program delivery via VR for Veterans with SCI following the M-PACE process. To reduce power asymmetry during deliberation, the committee will follow a modified Nominal Group Technique (58). All members will independently generate initial thoughts on each proposed adaptation, then share verbal pros and cons in a fixed round-robin order before open discussion, ensuring all perspectives including those of Veteran members are heard first. The team will use majority consensus, submitted privately and asynchronously via email after each meeting, to determine whether an adaptation is acceptable. The goal is to modify the clinical materials to optimize content and processes that are important, feasible, and congruent with stakeholder priorities. When consensus is not met, no change will be made. Written responses, round-robin input, and voting outcomes will be documented for each decision, alongside formal FRAME documentation of the rationale for accepted or rejected modifications.

At the completion of Phase 1, a modified VA CALM curriculum (VA CALM-SCI) will be generated. This adapted curriculum will provide recommendations for facilitators and participants to improve accessibility and appropriate fit for Veterans with SCI. This process involves evaluating participant feedback for *importance* to the enhancement of acceptability, *feasibility*, and *congruence* to core components of the program. After evaluating each suggestion, the steering committee will collectively determine whether to accept a proposed adaptation, with careful documentation of modifications and rationale. Program modifications will be recorded formally, using the FRAME (47).

#### 2.2.5 Phase 1 data analysis

Quantitative measures will be analyzed for exploratory purposes with means and standard deviations examined descriptively. Assessments in this phase will be used to establish the feasibility of the measure battery and to identify which measures are most appropriate and sensitive to meaningful change for a future RCT for efficacy testing of the fully adapted VA CALM-SCI.

Qualitative data will be used to guide adaptations as they provide nuanced insights on needs, program sticking points, and other contextual issues. Transcripts will be evaluated for key domains reflecting both predetermined (e.g., acceptability, feasibility, recommended modifications) and expected themes (e.g., contextual factors that may impact program workflow) to facilitate program modifications. The research team will use inductive and deductive coding to conduct both a content (59) and thematic analysis (60). A codebook for deductive analysis will be developed, using relevant constructs from the expanded FRAME (47), with open coding used to capture unexpected insights as the team analyzes data.

Coders will read and reread transcripts and supporting documents (field notes and post facilitation reflections) to familiarize themselves with the data, summarizing notes to triangulate key points across sources. Next, coding will begin, using the codebook developed deductively. After an initial pass, coders will open code for unanticipated concepts and themes. Next, they will finalize a code book, resolving disagreements through consensus and using a qualitative expert for tie breaking. Coders will then double code all transcripts and organize data by themes. Findings will be presented to the steering committee for formal adjudication of modifications.

### 2.3 Phase 2: VR feasibility and acceptability study

#### 2.3.1 Participants

Participants from Phase 1 will be invited back for Phase 2, the VR feasibility and acceptability testing to provide insights on the use of VR to provide this content. Participants will experience the newly modified curriculum in the VR setting to assess whether modifications were sufficient to address identified needs. If attrition occurs, we will use the same process of recruitment used in Aim 1 to recruit new participants to ensure the VR phase has sufficient Veteran input.

#### 2.3.2 VR setup and materials

VR Hardware: Meta Quest 3s VR headsets are affordable, commercially available VR headsets that provide immersive experiences without complex setup or the need for peripheral devices to be used in a home setting. Devices will be configured in developer mode, with commercially available applications side-loaded via a free mobile device management process due to budgetary constraints. This approach allows the study team to apply deployment and device settings without requiring individual participants to maintain personal consumer accounts.

The widespread availability of this device means a range of compatible aftermarket accessibility add-ons like halo head straps and grip straps for comfort and stability will be available for evaluation by the steering committee to develop recommendations for accommodations. Recognizing that VR controls may pose challenges for those with upper-extremity impairments, the steering committee will evaluate hands-free adaptive interaction options (built-in accessibility features (e.g., voice command, hand tracking, seated modes, etc.) and adaptive input hardware (e.g., sip-and-puff switches, adaptive mounts) that enhance accessibility.

VR Application: In Phase 2, the steering committee will explore commercially available VR meeting applications to identify and select the single application that best facilitates the delivery of VA CALM for SCI curriculum The committee will evaluate applications for functionality as a group meeting interface, user accessibility features, customizability, and potential for facilitation of mindfulness group meetings (i.e., availability of visual/auditory content that promotes presence immersion and mindfulness).

#### 2.3.3 Delivery of VA CALM-SCI modules via VR

In Phase 2, two modified VA CALM-SCI modules will be delivered to Veterans returning from Phase 1 to obtain feasibility and acceptability of using VR to deliver this content while also gathering feedback on the modified modules to assess appropriateness of modifications. The group facilitator will administer the modified curriculum in real time via a VR-based group setting, lasting approximately 60–90 min. The facilitator will follow the adapted curriculum guide during the synchronous/structured VR use class time, ensuring all participants receive the same content; and participants will be encouraged to use the VR application for mindfulness practice between sessions, with this asynchronous/non-structured VR use captured for feasibility.

Two modules were selected for the VR feasibility phase rather than the full 9-module curriculum due to feasibility of the funding mechanism. Earlier versions of the protocol proposed a more robust testing for this aim, but it was not deemed feasible for the award period. Module selection will be conducted by the steering committee and informed by findings from Phase 1. Modules, content, and activities requiring substantive adaptation for accessibility will be given priority to elicit valuable feedback on the changes made. This approach ensures that the limited VR development resources available in this formative phase are directed toward the modules for which accessibility barriers and, therefore, adaptation needs, are greatest, and for which VR delivery is most likely to yield meaningful feasibility and usability data to inform a future full-curriculum trial.

#### 2.3.4 Data collection

##### 2.3.4.1 Quantitative

In Phase 2, quantitative data will focus on the feasibility, acceptability, usability, and embodiment of VR. The feasibility, acceptability, and usability measures will inform refinement of the technical portions of the program, while the embodiment measures inform the utility of VR in facilitating presence during the mindfulness sessions. Table 4 includes descriptions of the measures used in Phase 2 and collection time points.

**Table 4:** Phase 2 quantitative measures and collection timelines.

Outcome	Measure	Length and est. completion	Collection timepoint
Primary outcome			
Feasibility of VR	Session attendance; assessment completion; program completion rates; time in VR (session time and voluntary time)		–
Secondary outcomes			
Acceptability of VR delivered program	Acceptability of Intervention Measure (AIM) (63) Intervention Appropriateness Measure (IAM) (63)	4 items, 1 min. 4 items, 1 min.	Completion of 2nd VR session
VR usability	System Usability Scale (SUS) (61) Standardized User Experience Percentile Rank Questionnaire (SUPRQ) (64)	10 items, 3 min. 8 items, 2 min.	Completion of 2nd VR session
VR embodiment	One-Item Presence Scale (65) Virtual Embodiment Questionnaire (66)	1 item, 1 min. 12 items, 3 min.	

Predefined feasibility and acceptability benchmarks for Phase 2 include: completion of both VR sessions by at least 80% of returning participants; a mean System Usability Scale (SUS) score of ≥70, the standard threshold denoting “above average” usability (61); mean Acceptability of Intervention Measure (AIM) and Intervention Appropriateness Measure (IAM) scores above the scale midpoint (62); and no more than 1 VR-related adverse event. These criteria will guide go/no-go decisions for advancing to a fully powered pilot and RCT.

##### 2.3.4.2 Qualitative data

In Phase 2, qualitative data will inform practical and contextual information related to creating a feasible, accessible, and acceptable VR experience for participants. Following the same qualitative approach in Phase 1, the team will use field notes, reflections, and a final post-VR focus group to inform program refinement. Table 5 provides a description of data sources and collection points.

**Table 5:** Phase 2 qualitative data and collection timelines.

Source	Description	Collection timepoint
Field notes	Documentation of content, context, and concepts pertaining to participant needs and program implementation (e.g., accessibility issues, etc.)	After each VR session
Facilitator reflections	Documentation of facilitator impressions and observations related to program content and delivery	After each VR session
Focus groups	Semi-structured, open-ended questions, assessing content, delivery, and acceptability of the program	Completion of 2nd VR session

#### 2.3.5 Data analysis

Data analysis for both quantitative and qualitative data in Phase 2 will follow the same format as Phase 1. Findings from Phase 2 will provide insights on feasibility, usability, acceptability, and the potential benefits and challenges of using VR to deliver mindfulness programming. The team will also use focus groups to gather feedback on the modifications made to program content to determine whether modifications meet the needs of program participants and/or whether additional modifications are needed.

### 2.4 Ethics approval

This study protocol been approved by the Institutional Review Board at Baylor College of Medicine and the Research & Development Committee at the Michael E. DeBakey Veterans Affairs Medical Center (Protocol Number H-55733) and is registered with the National Library of Medicine ClinicalTrials.Gov (NCT06587607). All participants in the study will be consented in writing prior to starting study activities.

## 3 Discussion

The objective of this study is to explore the feasibility and acceptability of a VR delivered, SCI-tailored, evidence-based mindfulness group (VA CALM) using a mixed-methods approach. However, given the complex needs of individuals with SCI, development of an accessible and disability-centered approach is needed to ensure application, content, and systems used fit the needs of this population. This approach seeks to address unintended inequities in digital health offerings as they pertain to accessibility, acceptability, and content fit. Using the M-PACE process allows the study team to develop appropriate adaptive strategies and accommodations while exploring the potential added benefits of using VR to facilitate mindfulness practice.

### 3.1 Expected contributions

At the completion of this study, we anticipate having a fully developed SCI-tailored accommodations guide to accompany the formal VA CALM program materials for VA CALM facilitators to use as needed when working with individuals who have motor and sensory impairments. We also expect to learn more about how VR can be used to facilitate immersion during mindfulness training, exploring the utility of using VR as a delivery platform for clinic-to-home treatments, and understand whether group clinic to home VR modalities are desirable and feasible for this type of intervention. By gathering user experience insights as well as expert feedback throughout this process, we anticipate that this guide may serve other populations with altered sensory and motor function (e.g., stroke). The results of this study will also provide preliminary data and feasibility for a future trial to determine the efficacy of this program, with lessons learned for other populations and treatments.

### 3.2 Strengths

Strengths of this study include the use of an established implementation science framework, M-PACE, which includes an interdisciplinary steering committee for the co-design process throughout the process to develop accommodations and adaptive strategies to enhance program fit and accessibility. Additionally, the use of a systematic mixed-methods approach provides valuable insights in program planning, while also balancing fidelity of the intervention with population context (62).

### 3.3 Limitations

Limitations of this study include limited generalizability due to the small sample size, which is appropriate at this stage of treatment adaptation, and recruitment from a single site. Additionally, the use of commercial VR platforms, while feasible, limits the ability of the team to customize software and may impact device/application usability for this population. However, these findings will provide valuable insights for future work by identifying essential benefits or gaps in commercially available applications to guide future recommendations and development of accessible applications for this purpose.

Furthermore, several exclusion criteria, while methodologically necessary for this formative phase, may limit the generalizability of our findings. For example:

- High-speed internet access is required because both the telehealth-delivered VA CALM modules in Phase 1 and the VR-delivered adapted modules in Phase 2 are delivered in a real-time, synchronous group format; without a stable connection, participants cannot reliably attend sessions.
- Basic computer literacy (e.g., the ability to open and respond to email) is necessary because the primary aim of this phase is to gather detailed, nuanced participant feedback to inform curriculum adaptation; participants unable to engage with basic study logistics and communication would be unable to fully contribute to this feedback process, undermining the study's core objective.
- Ability to use VR equipment at home, independently or with caregiver assistance, reflects the same rationale: this formative phase is designed to evaluate feasibility, acceptability, and usability of a clinic-to-home delivery model; and participants unable to use the technology outside a clinical setting would not generate data relevant to that specific aim.
- Ability to speak clearly and independently during focus groups reflects the substantial reliance of this phase on rich, nuanced qualitative feedback to guide the extensive content and process modifications needed to adapt VA CALM for this population.

These criteria, while appropriate for this initial adaptation work, exclude Veterans with more severe motor, sensory, communication, or cognitive impairment who may stand to benefit most from accessible digital interventions. This is a limitation we intend to directly address in subsequent phases of this work that would allow broader inclusion in future iterations and in a subsequent RCT.

Another limitation of this work is related to the evaluation of only 2 of the 9 adapted modules in VR. This scope was determined by the funding mechanism supporting this work. As such, feasibility and usability issues may not be fully captured. This study is intended to generate preliminary process and outcome data to support a subsequent, fully powered pilot and RCT of the complete VR-translated curriculum.

## 4 Conclusions

This study protocol describes a structured, disability-centered process for adapting an evidence-based mindfulness intervention to the needs of Veterans with SCI and for evaluating the feasibility and acceptability of its delivery through VR. By combining participatory co-design with an accessible VR modality, this work aims to reduce long-standing barriers to engagement in behavioral health interventions among individuals with complex motor and sensory impairments. Findings from this formative evaluation will guide refinement of both the adapted VA CALM-SCI curriculum and the technical implementation strategy, laying the groundwork for a future trial comparing VR-based delivery with video telehealth. Ultimately, this research seeks to broaden access to engaging, effective, and inclusive digital health solutions for Veterans with SCI and potentially other populations with mobility or sensory limitations.
