# Supplementary material for: Co-designing accessible digital mindfulness for veterans with spinal cord injury: protocol for developing and testing VA CALM-SCI for virtual reality
Source: Front Digit Health. 2026 Oct 8;8:1923501. doi: 10.3389/fdgth.2026.1923501 (PMC13688874; doi:10.3389/fdgth.2026.1923501)
Supplement: Supplementary file 1 [file Supplementaryfile1.pdf]

Supplemental Materials

Sample Field Notes Template

Date:

Session #:

Participant Names:

Context- Consider circumstances- Who is there as observer, what is your reason for being there, why this location, what is your state of min, what are the key areas of observational interest?

Content- Consider the substance of what happened- Who are the participants; how are they related? How do participants interact? What actions/events are happening? What is the timing/sequence? What quotes best capture the exchange that occurred?

Concepts- Consider the larger theoretical context (eg, future directions for research)- What have you learned that you didn't know? Does this observation help support or refute expectations? How does this observation relate to prior observations? What are some potential implications? What new questions arise? How do participants respond to the presence of the observer?

Program Content:

Keep

-
-
-

Modify

-
-
-

Program Technical:

Barriers

-
-
-

Facilitators

-
-
-

Program Accessibility:

Barriers

-
-
-

Facilitators

-
-
-

Sample Post-Session Facilitator Guide

Date:

Session #:

1. Overall, how did you feel today's session went?
2. Briefly describe today's activity.
3. What do you think the participants thought?
4. What aspects did they like/ understand/ engage in?
    a. Probe: What was the most successful aspect of the class? What was the least successful? Why? What would help address these issues?

Sample Focus Group Interview Guide

Background:

Thank you for volunteering your time to have a conversation with us today. We'd love to hear more about your experiences so far with the VA CALM. Your insight will advance our initiative in formulating creative solutions to virtual care for Veterans with Spinal Cord Injuries (SCI) experiencing chronic pain.

We are interested in a few components of your care, including the content delivery, technical complexities, and feasibility of applying this program. For each of these components, I will ask you a few questions; and please feel free to be as honest as possible. This will allow us to appropriately evaluate and update the program. Our conversation should last about 2 hours and will be held confidential. To make accurate analyses, we'd like to audio-record and transcribe this interview. If you give your consent to participate and to audio record and transcribe the interview, please state your name and your agreement to participate.

Content (to be asked at Focus Group meetings 1 & 2):

1. Overall, how would you describe your experience with the VA CALM Mindfulness program?
    - Probe: Try to focus on thoughts on program content (not VR modality; we'll ask about that later).
2. How well do you think the content was able to address your needs?
    - Probe: Were there sessions that you valued or connected with more? Why those sessions?

    What were your needs during this program? What parts met your needs? What parts failed to meet your needs? How could we change things to make them meet your needs?
3. How do you feel about the program structure (eg. 9 sessions, meeting 1x a week, group format, etc.)?
    - Probe: How convenient was it for you to adhere to the structure? Was it too long/too short? Too frequent/infrequent? What about the overall length of the program (9 sessions); was this too long/too short? Are there any additional supports you would like to see outside "class time" before starting the program, during the program, or after program completion?
4. How well does VA CALM meet the needs of other Veterans with SCI/chronic pain?
    - Probe: What do you think this modality can accomplish for others? What changes (if any) are needed to make this program work for Veterans with SCI? Would you recommend this program to other people? Why or why not?

Delivery (To be asked at Focus Group meeting 3):

5. How do you feel about using VR to deliver this intervention?
    - Probe: What was the experience with VR like? What did you like about it? What did you dislike about it? What could we do to make your experience better? How was it different than other methods, such as in-clinic visits or telehealth appointments?
6. Did you feel the program was effective through VR?
    - Probe: How well were you able to connect with the clinician? Other Veterans? Understand/connect with the information?
7. Did you have any complications with using VR?
    - Probe: What were the barriers and how did they affect you? How did this impact your participation? What did you do to deal with these barriers? How could we help others navigate these issues better?
8. How helpful was the training we provided when getting started with VR?
    - Probe: What aspects of the training were helpful, unhelpful? What do you wish should be added/removed?
9. How do you feel these complications can be eliminated or bridged for the overall program?
    - Probe: What suggestions do you recommend on how technical issues can be alleviated or bridged?

Feasibility/Acceptability (To be asked at Focus Group meeting 3):

10. How would you compare other modalities with VR for healthcare appointments?
    - Probe: Consider other modalities, in-person, video telehealth, telephone, etc.
11. What are your thoughts on further developing VR for mindfulness training for Veterans with SCI and chronic pain?
    - Should it be continued/discontinued – why?
12. What influence did receiving this program in VR have on you?
    - Probe: How do you feel now that you are done with the program?
13. Would you consider using VR again in the future?
    - Probe: For any concern - mindfulness, therapy appointments, entertainment, etc.? Why or why not?
14. How do you anticipate using VR for this program will affect other Veterans with SCI/chronic pain?

    Probe: What do you think this modality can accomplish for others? Would you recommend this program to other people? Why or why not?
